# O094: Avatar approach on hand hygiene

**DOI:** 10.1186/2047-2994-2-S1-O94

**Published:** 2013-06-20

**Authors:** BS Ana, C Palos

**Affiliations:** 1 Infection Control Comittee, Hospital Beatriz Ângelo, Loures, Portugal

## 

This videoclip aims to introduce visitors and patients on the hospital hand hygiene policy and procedures as well as a reminding for professionals on daily basis. The video explains in a simple and attractive way, how to proceed, using a combination of avatar technology and real persons.SPECIAL; SPECIAL;Videoclips are a part of a global awareness and teaching materials on Infection Control and Prevention, available on hospital intranet and on infection control committee sessions.SPECIAL; SPECIAL.

## Disclosure of interest

None declared.

